# Salivary Lactoferrin Expression in a Mouse Model of Alzheimer’s Disease

**DOI:** 10.3389/fimmu.2021.749468

**Published:** 2021-09-30

**Authors:** Desiree Antequera, Diego Moneo, Laura Carrero, Fernando Bartolome, Isidro Ferrer, Gordon Proctor, Eva Carro

**Affiliations:** ^1^ Network Centre for Biomedical Research in Neurodegenerative Diseases (CIBERNED), Alzheimer’s Disease and Other Degenerative Dementias, Madrid, Spain; ^2^ Group of Neurodegenerative Diseases, Hospital Universitario 12 de Octubre Research Institute (imas12), Madrid, Spain; ^3^ Bellvitge Biomedical Research Institute (IDIBELL), Hospitalet de Llobregat, Barcelona, Spain; ^4^ Department of Pathology and Experimental Therapeutics, University of Barcelona, Hospitalet de Llobregat, Barcelona, Spain; ^5^ Institute of Neurosciences, University of Barcelona, Barcelona, Spain; ^6^ Centre for Host Microbiome Interactions, King’s College London, London, United Kingdom

**Keywords:** lactoferrin, saliva, submandibular glands, Alzheimer’s disease, acetylcholine, muscarinic receptors, immune system, antimicrobial protein

## Abstract

In the last few years, microbial infection and innate immune theories have been proposed as an alternative approach explaining the etiopathogenesis and origin of Alzheimer’s disease (AD). Lactoferrin, one of the main antimicrobial proteins in saliva, is an important modulator of immune response and inflammation, and represents an important defensive element by inducing a broad spectrum of antimicrobial effects against microbial infections. We demonstrated that lactoferrin levels in saliva are decreased in prodromal and dementia stages of AD compared with healthy subjects. That finding seems to be specific to cerebral amyloid-β (Aβ) load as such observation was not observed in healthy elderly controls or those subjects with frontotemporal dementia. In the present study, we analysed salivary lactoferrin levels in a mouse model of AD. We observed robust and early reduction of lactoferrin levels in saliva from 6- and 12-month-old APP/PS1 mice. Because saliva is secreted by salivary glands, we presume that deregulation in salivary glands resulting in reduced salivary lactoferrin levels may occur in AD. To test this hypothesis, we collected submandibular glands from APP/PS1 mice, as well as submandibular gland tissue from AD patients and we analysed the expression levels of key components of the salivary protein signalling pathway. A significant reduction in M3 receptor levels was found along with decreased acetylcholine (Ach) levels in submandibular glands from APP/PS1 mice. Similarly, a reduction in M3 receptor levels was observed in human submandibular glands from AD patients but in that case, the Ach levels were found increased. Our data suggest that the ACh-mediated M3 signalling pathway is impaired in salivary glands in AD, resulting in salivary gland dysfunction and reduced salivary lactoferrin secretion.

## Introduction

Alzheimer’s disease (AD) is a progressive neurodegenerative disease with key pathological hallmarks including amyloid plaques, neurofibrillary tangles, and neurodegeneration (1, 2). Although Aβ and tau hyperphosphorylation plays a central role in the development of AD, increasing evidence suggests that AD pathogenesis includes strong interactions with immune mechanisms in response to brain infections (3).

In the last few years, an additional explanation for the etiopathogenesis of AD has been proposed, involving potential microbial infections along with an innate immune origin for the disease. A number of studies show strong evidence proving the presence of microbial infectious in AD brains, including viruses, bacteria and fungi (4–11). AD is a complex disease and microbial infection may be one of the disease mechanisms triggering the pathology in which an interplay between genetic and other environmental risk factors along with the infections contribution and the immune response exists (12).

The innate immune system uses antimicrobial peptides and proteins against invading microorganisms or pathogens (13). In the last decade, several studies explored a novel role for antimicrobial peptides in AD pathology as potential biomarkers (14). One of the most promising antimicrobial proteins may be lactoferrin (14, 15). Lactoferrin is a multifunctional iron-binding glycoprotein, found abundantly at mucosae, and in secreted fluids, including saliva, with a potent antimicrobial and immunomodulatory activity (16, 17). Lactoferrin represents an important defensive element by inducing a broad spectrum of antimicrobial effects against bacteria, fungi, protozoa, viruses and yeasts (18). It plays an important role in regulating the oral microbiota and the inflammatory state of the oral mucosa (19) and contributes to maintaining the symbiosis in the host-microbiome relationship in AD (20). Additionally, other physiological functions of lactoferrin include antioxidant activities, neuroprotection, immune response regulation, anti-inflammatory and anti-carcinogenic potential (21–25).

In 2017 we demonstrated a reduction in salivary lactoferrin levels in AD patients as well as patients with mild cognitive impairment (MCI), when compared with healthy controls (26). More recently we have shown that reduced salivary lactoferrin levels correlate with increased cerebral amyloid-β (Aβ) and this observation is specific to MCI and AD patients but not to healthy elderly controls or patients with frontotemporal dementia (27). Therefore, we proposed that the reduced lactoferrin levels in AD could reflect compromised immunity that may result in an enhanced risk of brain infections (28).

Pre-clinical models meeting the main pathological hallmarks of AD, including amyloidosis, are very useful to study the AD mechanisms of pathology. Moreover, well-characterised genetically-induced mouse models avoid the diagnostic uncertainty and interfering comorbidities that are present in patients. Here, we studied the regulation of salivary lactoferrin levels in a mouse model of cerebral amyloidosis (APP/PS1 transgenic mice). We observed an early and robust reduction in salivary lactoferrin levels in APP/PS1 mice at 6-, and 12-month-old. Saliva and salivary proteins are secreted from salivary glands under the autonomic nervous system control. Particularly, through acetylcholine (Ach) action on the acinar M3 muscarinic receptors in salivary glands, the parasympathetic nervous system stimulates the saliva secretion (29–31). Based on these findings, we presumed that deregulation in salivary glands may occur in AD. To test this hypothesis, we collected submandibular glands from APP/PS1 mice as well as submandibular gland tissue from AD patients. We then analysed the M3 muscarinic receptor and Ach levels, both regulating the salivary lactoferrin expression and levels. We found a significant reduction in M3 receptor and Ach levels in submandibular glands from APP/PS1 mice. Human AD submandibular glands showed a similar reduction in M3 receptor expression but increased Ach levels.

## Methods

### Animal Experiments

Male double transgenic APP/PS1 mice (6- and 12-month-old), a cross between Tg2576 (overexpressing human APP695) and mutant PS1 (M146L), were used from our inbred colony (Instituto de Investigación Hospital 12 de Octubre). Age-matched mice not expressing the transgene were used as wild-type (WT) controls. Animals were sacrificed by deep anaesthesia and perfused transcardially either with saline for biochemical analysis, or 4% paraformaldehyde (PFA) in 0.1 M phosphate buffer (PB), pH 7.4 for immunohistochemical analysis. All animals were handled and cared for according to the Council Directive 2010/63/UE of 22 September 2010 and ARRIVA guidelines (2020). Sample size was estimated for each condition using the SPSS statistics software, assuming a normal distribution in all cases. This sample size is necessary to minimise the number of animals obtaining significant differences between groups and reducing the possible intragroup variability with an established statistical power of 95% confidence. In all cases, variability or standard deviation lower than the mean along with less than 10% of losses (mice deaths) are expected.

### Saliva Collection

APP/PS1, and WT mice were anaesthetised by isoflurane inhalation and subcutaneously injected with pilocarpine (0.5 mg/kg i.p.; Novartis Farmacéutica, S.A. Barcelona, España) to induce salivation. Saliva collection proceeded for 5-10 min following stimulation with pilocarpine, collected into Eppendorf tubes from each mouse, and stored at -80°C.

### Human Submandibular Glands

Post-mortem submandibular gland samples were obtained from brain donors diagnosed with AD and healthy control individuals. Frozen samples were supplied by the Institute of Neuropathology Brain Bank IDIBELL-Hospital Universitari de Bellvitge (Hospitalet de Llobregat, Spain), the University of Alabama at Birmingham-CHTN (USA), and Banner Sun Health Research Institute, Arizona (USA). Subjects’ consent was obtained according to the Declaration of Helsinki, and approval came from the Research Ethics Committee of the above-mentioned responsible institutions. A total of 26 samples were categorised into two groups, as it is presented in **Supplementary Table 1**.

### Western Blot

Proteins were isolated from submandibular gland samples by standard methods. Briefly, submandibular glands were homogenised in lysis buffer NP-40 (50 mM Tris-base pH 7.4, 150 mM NaCl, 0.5% Nonidet P-40, 1 mM EDTA) containing a mixture of protease and phosphatase inhibitors (ROCHE cOmplete™ Protease Inhibitor Cocktail), and centrifuged for 15 min at 10000 rpm at 4°C. The supernatant was recovered and stored at -80°C. Protein estimation from salivary gland lysates and saliva samples was determined using the BCA assay (Pierce BCA Protein Assay Kit, Thermo Fisher, Waltham, MA, USA). Each sample was loaded in a precast 10% Tris-HCl (CriterionTM TGX Stain-FreeTM Precast Gels, BioRad Laboratories, CA, USA) and the separated proteins were transferred to polyvinylidene fluoride (PVDF) membranes (BioRad Laboratories). Primary antibodies utilised were: rabbit polyclonal anti-lactoferrin antibody (ab135710, Abcam Cambridge, UK), and rabbit polyclonal anti-M3mAChR antibody (ab126168, Abcam Cambridge, UK). Protein loading was monitored using a mousemonoclonal antibody against β-actin (A1978, 1:10000; Sigma-Aldrich, St. Louis, USA). Membranes were then incubated for 1 hour with the appropriate horseradish peroxidase-conjugated secondary antibodies (1:2000; Dako, CA, USA), and immunocomplexes were revealed by an enhanced chemiluminescence reagent (ECL Clarity; BioRad Laboratories). Densitometric quantification was carried out with Image Studio Lite 5.0 software (Li-COR Biosciences, NE, USA). Protein bands were normalised to β-actin levels and expressed as a percentage of the control group.

### Immunohistochemistry

Submandibular glands tissue was fixed for 24 hours in 4% paraformaldehyde (PFA) by immersion. Then, submandibular gland samples were paraffin-embedded for subsequent cryotome sectioning (Leica, Wetzlar, Germany). 4 µm thick sections were processed free-floating for immunohistochemistry. All primary antibodies were diluted in PB 0.1 M containing 0.5% bovine serum albumin and 0.5% Triton X-100. The following primary antibodies were used: rabbit antiserum anti-lactoferrin antibody (07-685, Merk Millipore, Darmstadt, Germany), After overnight incubation, primary antibody staining was revealed using the avidin-biotin complex method (VECTASTAIN Elite ABC Kit, Vector Laboratories, Burlingame, CA, USA). The sections were counterstained with Vector haematoxylin (H3401, Vector Laboratories). Images were captured using a light microscope (Zeiss microscope; Carl Zeiss Microimaging, GmbH, Oberkochen, Germany). All images were analysed using the Volocity software (PerkinElmer, Waltham, MA, USA).

### RNA Extraction and Quantification

RNA was obtained from APP/PS1 and WT submandibular glands using NZYol (NZYTech, Lda., Lisboa, Portugal) following the manufacturer’s protocol. RNA concentration was measured in a NanoDrop™ One Spectrophotometer (ThermoFisher) and 1µg of each sample was retrotranscribed to cDNA using iScript™ cDNA Synthesis Kit (Bio-Rad). Quantitative real-time PCR (qRT-PCR) was performed in a LightCycler^®^ 480 Instrument (Roche Diagnostics) using NZYSpeedy qPCR Green Master Mix (NZYTech, Lda., Lisbon, Portugal) and different oligonucleotides: lactoferrin and the housekeeping gene (*HPRT*) (**Supplementary Table 2**).

Relative levels of mRNA were calculated using crossing-point (Cp) data and ΔΔCp method (also known as ΔΔCt). Cp data from the gene of interest (GOI) were normalised to mean of endogenous gene *HPRT* data to obtain ΔCp data (ΔCp = mean Cp*_HPRT_* - Cp*_GOI_*). ΔΔCp was calculated between the normalised ΔCp values from each time point.

### Acetylcholine Determination

The amount of acetylcholine (ACh) from mice submandibular tissue was determined using a commercially available choline/ACh assay ELISA kit (Abcam, Cambridge, UK) according to the manufacturer’s instructions.

### Data and Statistical Analysis

All data are expressed as the mean ± standard error of the mean (SEM) in percentage. Sample size estimation was carried out using the IBM SPSS Statistics software, Version 21.0. (Armonk, NY, USA). Statistical analysis and exponential curve fitting were performed using GraphPad Prism 6.01 (GraphPad Software, La Jolla, CA, USA) software. Statistical analysis was carried out using Student’s t-test for two group comparisons. For more than two group comparisons the one-way Analysis of variance (ANOVA) followed by Tukey’s correction test was used. Statistical significance was set at p < 0.05.

## Results

### Lactoferrin Levels Are Decreased in Saliva From APP/PS1 Mice

**Figure 1:**
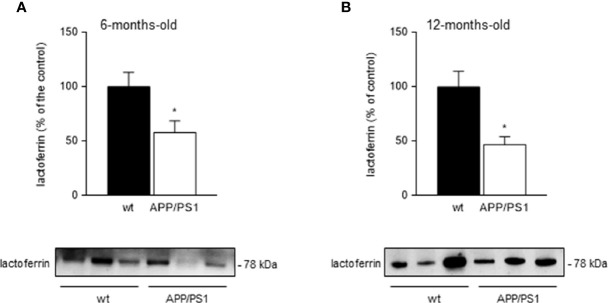
Salivary lactoferrin levels in APP/PS1 and WT mice. **(A, B)** Western blot analysis showed that lactoferrin levels were reduced in the submandibular gland samples from **(A)** 6- and **(B)** 12-month-old APP/PS1 compared with WT mice (*n* = 8-15, per group). Representative Western blots (lower panels) and histograms with their densitometric analysis (upper panels) are shown. Data are represented as mean ± SEM. Differences between groups were assessed using the Mann-Whitney test. *p < 0.05.

We tested salivary lactoferrin levels in 6- (initial-moderate symptoms) and 12 month-old (severe symptoms) APP/PS1 and WT mice by western blot using 4µl of saliva. We found that salivary lactoferrin levels were significantly lower in 6- (p < 0.05; **Figure 1A**) and 12- (p < 0.05; **Figure 1B**) month-old transgenic APP/PS1 mice compared with age-matched WT mice. These results were consistent with our previous studies carried out in patients (26, 27) showing that lactoferrin levels determined by ELISA were reduced in saliva from MCI and AD patients. Interestingly, total protein secretion into saliva showed no changes in APP/PS1 mice compared with WT mice either at 6- or 12-month-old (4.03 ± 0.41 µg/ml *vs* 4.10 ± 0.30 µg/ml, and 2.94 ± 0.60 µg/ml *vs* 2.10 ± 0.35 µg/ml, respectively, p > 0.05). Therefore, we may suggest that salivary lactoferrin levels in APP/PS1 mice progressively decrease starting at the early stages of neurodegeneration.

### Lactoferrin Expression in Submandibular Glands From APP/PS1 Mice Remained Unaltered

**Figure 2:**
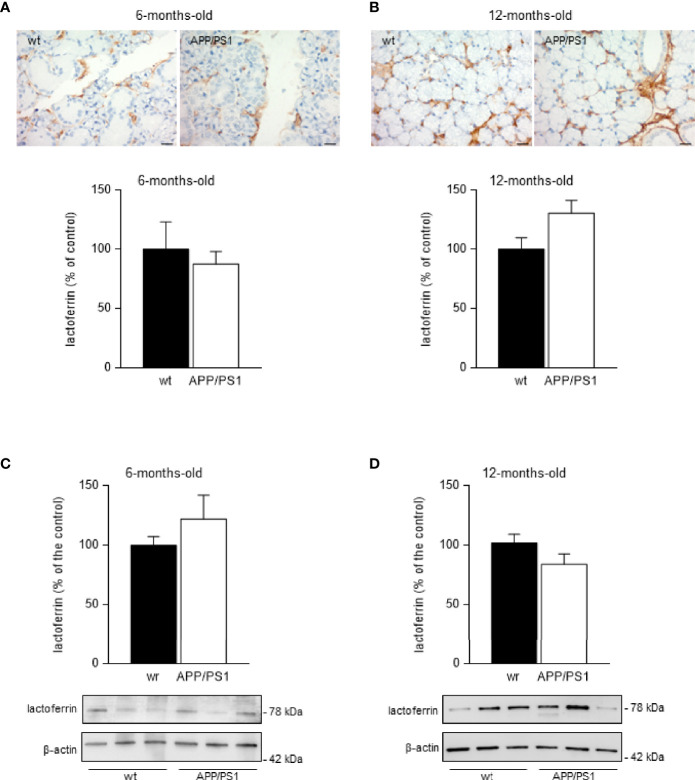
Lactoferrin levels in mice submandibular glands. **(A, B)** Lactoferrin stained (brown) sections of submandibular glands from **(A)** 6- and **(B)** 12-month-old APP/PS1 and WT mice. Size bar 20 μm. All sections were counterstained with haematoxylin (blue). In the lower histograms, quantification of the lactoferrin signal from 6- and 12-month-old APP/PS1 and WT mice submandibular glands, respectively (*n* = 8, per group) is shown. **(C, D)** Western blot analysis showing lactoferrin levels in submandibular glands from **(C)** 6- and **(D)** 12-month-old APP/PS1 and WT mice (*n* = 12-18, per group). Representative Western blots (lower panels) and histograms with their densitometric analysis (upper panels) are shown. Data are represented as mean ± SEM. Differences between groups were assessed using the Mann-Whitney test.

Lactoferrin and other antimicrobial substances have been found in rat submandibular glands, particularly in serous acini and ductal cells (32). The identification of cellular compartments *via* immunohistochemistry may help to understand the role and function of the submandibular glands. Therefore, we performed an immunohistochemistry analysis on submandibular glands from 6- and 12- month-old APP/PS1 and WT mice. As expected, robust lactoferrin immunoreactivity at the plasma membrane of submandibular acinar cells was observed in submandibular glands from APP/PS1 and age-matched WT mice (**Figures 2A, B**). However, there were no clear differences in the staining intensity across 6- and 12-month-old mice groups. To confirm these results, we also analysed lactoferrin levels in submandibular glands from 6- and 12- mount-old APP/PS1 and WT mice by western blot. No differences were found between the mice groups (**Figures 2C, D**). Additionally, mRNA levels of lactoferrin showed no changes in submandibular glands from 6- and 12- month-old APP/PS1 and WT mice (data not shown).

### Muscarinic M3 Receptor Levels Are Reduced in Submandibular Glands From APP/PS1 Mice and AD Patients

**Figure 3:**
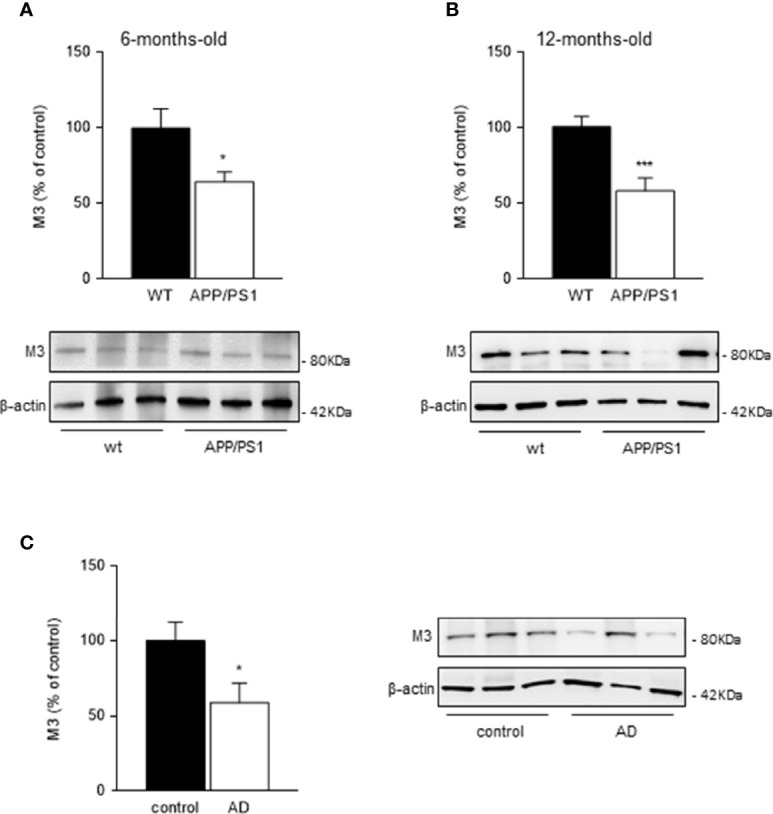
Muscarinic M3 receptor levels in mice and human submandibular glands. **(A, B)** Western blot analysis showed reduced M3 receptor levels in submandibular glands from **(A)** 6- and **(B)** 12-month-old APP/PS1 compared with WT mice (*n* = 8-13, per group). **(C)** In submandibular glands from AD patients, M3 receptor levels were also decreased compared with healthy control subjects (*n* = 8-10, per group). Representative Western blots and histograms with their densitometric analysis are shown. Data are represented as mean ± SEM. Differences between groups were assessed using the Mann-Whitney test. *p < 0.05; ***p < 0.001.

Since saliva secretion evoked by cholinergic stimulation is mediated by activation of muscarinic M3 receptors, we determined the levels of M3 receptors in submandibular glands from 6- and 12-month-old mice groups by western blot. We found a significant decrease in M3 receptor levels in submandibular glands from both 6- (p < 0.05; **Figure 3A**) and 12- (p < 0.001; **Figure 3B**) month-old transgenic APP/PS1 mice compared with age-matched WT mice.

M3 receptor levels in human submandibular glands showed equivalent results to those observed in mice. A significant reduction in M3 receptor levels in submandibular glands from AD patients compared with healthy control subjects was observed (p < 0.05; **Figure 3C**).

### ACh Content Is Differently Expressed in Submandibular Glands From APP/PS1 Mice and AD Patients

**Figure 4:**
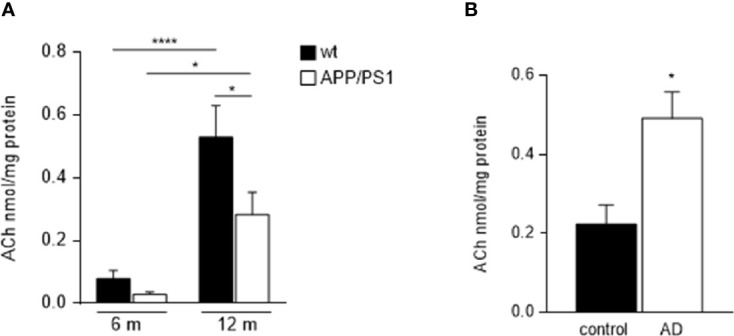
Ach levels in submandibular glands from APP/PS1 mice and AD patients. **(A)** Salivary gland ACh levels increased with ageing in both, APP/PS1 and WT mice. However, APP/PS1 mice showed lower ACh levels in their submandibular glands compared to WT mice, significantly at 12-month-old (*n* = 9-10, per group). **(B)** There were higher levels of ACh in submandibular glands from AD patients compared with control subjects (*n* = 8-9, per group). Data are represented as mean ± SEM. Differences between groups were assessed using the ANOVA and Tukey’s test for mice samples, and the Mann-Whitney test for human samples. *p < 0.05; ****p < 0.0001.

Due to the above-mentioned mechanism involved in salivary secretion, we suggest that changes in ACh levels in submandibular glands may result in the modification of salivary secretory function in AD pathology. Therefore, we evaluated ACh content in submandibular glands homogenates from 6- and 12-month-old mice groups. Although ACh levels were found increased in the 12-month-old compared with 6-month-old animals in both genotypes, (APP/PS1 = p < 0.0001, and WT = p < 0.05, respectively; **Figure 4A**), APP/PS1 mice showed lower ACh levels in their submandibular glands compared with WT mice, and this was significant at 12- month-old (p < 0.05; **Figure 4A**). These results along with the observed reduction in M3 receptor levels in submandibular glands from APP/PS1 mice (**Figures 3A, B**) suggest that dysfunction in the ACh-mediated M3 signalling pathway may cause impaired salivary gland function, leading to reduced salivary lactoferrin secretion.

On the contrary, ACh levels in the submandibular gland from AD patients were found higher compared with control subjects (p < 0.05; **Figure 4B**), in agreement with previously reported data in saliva (33). These findings may be explained by the use of acetylcholinesterase (AChE) inhibitors in the treatment of AD, which may help to increase and prolong the activity period of the released Ach. Supporting this hypothesis, decreased salivary AChE activity was reported in patients with AD compared with age-matched subjects, with significant differences in those who responded to AChE inhibitor treatment (34).

## Discussion

Here, we demonstrated that salivary lactoferrin levels are reduced in APP/PS1 compared with WT mice. This reduction was already detected at 6-month-old AD mice, equivalent to an initial stage of the disease, and such reduction remained at 12-month-old AD mice, equivalent to a moderate-severe stage. Our findings are consistent with our previous works examining human salivary samples, revealing that salivary lactoferrin levels are decreased in the prodromal and dementia stages of AD (26, 27). These data suggest that the APP/PS1 mice are suitable to model human salivary lactoferrin secretion. However, unexpectedly, lactoferrin content showed no changes in submandibular glands from these transgenic mice suggesting that the observed reduction in saliva was protein-synthesis independent. We further found that the muscarinic M3 receptor levels were decreased in submandibular glands from 6- and 12-month-old APP/PS1 mice compared with WT along with lower Ach levels. In addition, submandibular glands from AD patients showed similar reduction in M3 receptor levels but interestingly, increased Ach.

Saliva is an exocrine secretion containing different enzymes and antimicrobial proteins, along with immunoglobulins, growth factors, mucosal glycoproteins, and regulatory peptides (35, 36). One of these salivary antimicrobial proteins is lactoferrin, an important modulator of the immune response and inflammation (37–39). Lactoferrin represents an important defensive element by inducing a broad spectrum of antimicrobial effects against bacteria, fungi, protozoa, viruses, and yeasts (18). Salivary lactoferrin was found to decrease at the early stages of AD (26), therefore we proposed that deficiency of salivary lactoferrin in AD patients might facilitate oral bacterial or viral proliferation and the expansion of pathogens or their inflammatory products to the central nervous system (28).

Salivary glands actively secrete most of the buccal salivary components, including water, ions, and proteins. Such secretion depends on the autonomic nerve supply, particularly parasympathetic nerves, in a nerve-mediated reflex (29, 40). The primary parasympathetic salivary centres establish connections with the lateral hypothalamus. Hypothalamic-related deficits have been described in association with AD, even before the occurrence of any clinical symptoms of cognitive decline (41). Several hypothalamic nuclei showed pathological hallmarks including reduced neuronal population, dystrophic axons, abnormal Golgi, and synaptic spines. However, the characteristic AD pathology (neurofibrillary tangles and senile plaques) in these areas was absent in early-onset AD cases, suggesting that these morphological hypothalamic neuron alterations could appear very early in AD, prior to Aβ and tau deposition (42, 43). Several immunity deficits may occur during the AD preclinical stage and it is possible that they could facilitate AD progression according to experimental and clinical findings (44). Many authors suggest that immune system disturbances associated with AD may be the consequence of nervous system dysfunction determined by hypothalamic lesions (45, 46). We recently proposed that due to the dysregulation of hypothalamic immunity in early AD, salivary lactoferrin could be downregulated in AD, reflecting other disturbances of systemic immunity associated with this neurodegenerative disease (28).

Both, the volume and composition of secreted saliva depend on the autonomic nerve-mediated stimulation, particularly the parasympathetic system. Autonomic parasympathetic efferent nerves conduct signals from the salivary centres in the brain medulla to salivary glands, and salivary secretion is influenced by projecting neurons from the lateral hypothalamus to the salivary centres, which have excitatory or inhibitory effects on this signalling (40). Salivary secretion is largely dependent upon the activation of muscarinic receptors on salivary acinar cells through the release of ACh from parasympathetic nerves (40). There are five subtypes of muscarinic cholinergic receptors, M_1_–M_5_ (47, 48), but although some of them participate in the presynaptic and postsynaptic transmission (31), likely muscarinic M3 receptors are the principal mediators of the cholinergic response in salivary glands (31, 49). Salivary gland acinar cells secrete saliva following the interaction of ACh from parasympathetic nerves with M3 muscarinic receptors (30, 31, 49, 50). Therefore, it appears that the observed reduction in both M3 receptors and Ach levels in submandibular glands from APP/PS1 mice may deregulate salivary secretion, resulting in lower lactoferrin levels in the saliva fluid. However, no significant differences were previously found in the volume of saliva evoked by pilocarpine stimulation in APP/PS1 mice compared with their respective WT mice (51).

Our findings in human salivary gland samples partially differ from those obtained in a mouse model of AD. Although we found a significant reduction in M3 receptor levels in submandibular glands from AD patients compared with healthy control subjects, glandular Ach levels were found to increase. Specifically, Ach is the most studied neurotransmitter in AD and constitutes one of the most studied therapeutic targets (52). According to the cholinergic hypothesis (53), the main cause of AD is the reduction in ACh synthesis. Then, alterations in cholinergic activity may affect salivary production. In order to reduce the cholinergic conduction defect, AChE inhibitors are widely used in AD treatment as they help to increase and prolong the activity period of the released ACh. In agreement with this, AChE activity was found significantly lower in AD patients compared with healthy control age-matched subjects (34). Moreover, a recent study showed higher levels of ACh in saliva from AD patients (33). In our present study, 13 out of 15 AD patients were under AchE inhibitor treatment, which could explain the higher Ach levels observed in their submandibular glands. This partial recovery in the ACh-mediated M3 signalling pathway might improve salivary gland function, leading to enhanced salivary lactoferrin secretion. Supporting this hypothesis, we recently reported that salivary lactoferrin levels were significantly increased in AD patients under AChE inhibitor treatment (54).

Future studies conducted in age-matched cohorts will be critical to confirm our findings and will provide a more complete understanding of salivary dysfuntion in the AD pathology as the mean age of control group is significantly lower than in AD patients. Salivary gland alterations and dysfunction, including the reduced glandular and acini volume, along with the loss of secretory volume, are associated with different diseases but also with aging (55, 56).

Saliva plays an essential role in maintaining the equilibrium of the resident oral microbiota, providing antimicrobial activity through numerous proteins and peptides including lactoferrin, which blocks the adherence and colonisation of pathogens. Salivary gland hypofunction and altered salivary composition resulting in low salivary lactoferrin levels, often lead to perturbation of the function and composition of the oral microbiota causing dysbiosis, a plausible contributory factor in the development of AD pathophysiology (20, 57).

In summary, we may hypothesise that the low release of ACh and the subsequent decreased binding of ACh to M3 receptor might explain the impaired function of salivary glands. The consequence would be a reduced salivary lactoferrin secretion in AD patients. Our findings suggest that salivary lactoferrin may serve not only as a biomarker for AD but also as a potential biomarker and target for salivary gland dysfunction.

## Data Availability Statement

The raw data supporting the conclusions of this article will be made available by the authors, without undue reservation.

## Ethics Statement

The studies involving human participants were reviewed and approved by Institute of Neuropathology Brain Bank IDIBELL-Hospital Universitari de Bellvitge (Hospitalet de Llobregat, Spain) The University of Alabama at Birmingham-CHTN (USA) Banner Sun Health Research Institute, Arizona (USA). The patients/participants provided their written informed consent to participate in this study. The animal study was reviewed and approved by Autonomous University Ethics Committee for Animal Experimentation (license number: CEI 97 – 1778 – A291).

## Author Contributions

EC designed the study. DA, DM, and LC carried out the experimental analysis. IF provided salivary gland samples. FB, GP, and EC have reviewed and helped draft the manuscript. All authors contributed to the article and approved the submitted version.

## Funding

This study was supported by grants from Instituto de Salud Carlos III (FIS18/00118), FEDER, Comunidad de Madrid (S2017/BMD-3700; NEUROMETAB-CM), and CIBERNED (CB07/502).

## Conflict of Interest

EC is the co-founder of GEROA Diagnostics.

The remaining authors declare that the research was conducted in the absence of any commercial or financial relationships that could be construed as a potential conflict of interest.

## Publisher’s Note

All claims expressed in this article are solely those of the authors and do not necessarily represent those of their affiliated organizations, or those of the publisher, the editors and the reviewers. Any product that may be evaluated in this article, or claim that may be made by its manufacturer, is not guaranteed or endorsed by the publisher.
