# Supplementary material for: Salivary Lactoferrin Expression in a Mouse Model of Alzheimer’s Disease
Source: Front Immunol. 2021 Sep 30;12:749468. doi: 10.3389/fimmu.2021.749468 (PMC8514982; doi:10.3389/fimmu.2021.749468)
Supplement: Supplementary file 1 [file Table_1.docx]

**Suppl. Table 1.** Demographic and clinical data of participants.

|  | CONTROL | AD | P value |
| --- | --- | --- | --- |
| n | 11 | 15 |  |
| Sex (M/F) | 6/5 | 9/6 | ns |
| Age, mean (SD) | 62.27 (8.25) | 79.07 (8.01) | < 0.0001 |
| Age at onset, mean (SD) | - | 61.48 (10.7) | NA |
| Braak stage (n) | - | Braak II-III: 5  Braak VI: 10 | NA |
| MMSE score, mean (SD) | - | 12.8 (8.96) | NA |
| *APOE ε4* carrier, n (%) | - | 40 | NA |
| ChE inhibitor drugs, n | - | 13 | NA |

AD: Alzheimer’s disease; n: number; F: female. M: male; ns, non-significant; SD: standard deviation; NA, not applicable; MMSE: Mini-Mental State Examination; ChE: Cholinesterase.
