# Supplementary material for: Salivary Lactoferrin Expression in a Mouse Model of Alzheimer’s Disease
Source: Front Immunol. 2021 Sep 30;12:749468. doi: 10.3389/fimmu.2021.749468 (PMC8514982; doi:10.3389/fimmu.2021.749468)
Supplement: Supplementary file 2 [file Table_2.docx]

**Suppl. Table 2.** Real-time PCR oligonucleotides.

| Gene |  | Sequence (5'>3') |
| --- | --- | --- |
| m*Lft* | Fw  Rv | CAGGTTATGCTGGAGCCTTGA  GTACTGGTCCCTTTCGGCTTT |
| m*Hprt* | Fw  Rv | GTTGGGCTTACCTCACTGCT  TAATCACGACGCTGGGACTG |

*Lft*: lactoferrin; Fw: forward; Rv: reverse.
